# VERDICT‐AMICO: Ultrafast fitting algorithm for non‐invasive prostate microstructure characterization

**DOI:** 10.1002/nbm.4019

**Published:** 2018-10-31

**Authors:** Elisenda Bonet‐Carne, Edward Johnston, Alessandro Daducci, Joseph G. Jacobs, Alex Freeman, David Atkinson, David J. Hawkes, Shonit Punwani, Daniel C. Alexander, Eleftheria Panagiotaki

**Affiliations:** ^1^ UCL Centre for Medical Imaging London UK; ^2^ Department of Computer Science UCL Centre for Medical Image Computing London UK; ^3^ Computer Science Department University of Verona Italy; ^4^ Radiology Department Centre Hospitalier Universitaire Vaudois (CHUV) Switzerland; ^5^ University College London Hospitals London UK; ^6^ Department of Medical Physics UCL Centre for Medical Imaging Computing London UK

**Keywords:** AMICO, cancer imaging, diffusion MRI, microstructure imaging, prostate cancer, quantitative imaging, VERDICT MRI

## Abstract

VERDICT (vascular, extracellular and restricted diffusion for cytometry in tumours) estimates and maps microstructural features of cancerous tissue non‐invasively using diffusion MRI. The main purpose of this study is to address the high computational time of microstructural model fitting for prostate diagnosis, while retaining utility in terms of tumour conspicuity and repeatability.

In this work, we adapt the accelerated microstructure imaging via convex optimization (AMICO) framework to linearize the estimation of VERDICT parameters for the prostate gland. We compare the original non‐linear fitting of VERDICT with the linear fitting, quantifying accuracy with synthetic data, and computational time and reliability (performance and precision) in eight patients. We also assess the repeatability (scan‐rescan) of the parameters.

Comparison of the original VERDICT fitting versus VERDICT‐AMICO showed that the linearized fitting (1) is more accurate in simulation for a signal‐to‐noise ratio of 20 dB; (2) reduces the processing time by three orders of magnitude, from 6.55 seconds/voxel to 1.78 milliseconds/voxel; (3) estimates parameters more precisely; (4) produces similar parametric maps and (5) produces similar estimated parameters with a high Pearson correlation between implementations, *r* ^2^ > 0.7. The VERDICT‐AMICO estimates also show high levels of repeatability. Finally, we demonstrate that VERDICT‐AMICO can estimate an extra diffusivity parameter without losing tumour conspicuity and retains the fitting advantages.

VERDICT‐AMICO provides microstructural maps for prostate cancer characterization in seconds.

**Abbreviations used**
ADC: apparent diffusion coefficient
AMICO: accelerated microstructure imaging via convex optimization
CV: coefficient of variation (SD of mean values in %)
CZ: central zone
*d*_*i*_: diffusivity
DT: diffusion tensor
DW‐MRI: diffusion‐weighted MRI
EES: extracellular‐extravascular
*f*_*i*_: proportion of the signal/volume fraction
GPU: graphical processing unit
g‐ROI: grouped ROI
IC: intracellular
*N*_AV_: number of averages
*P*: pseudo‐diffusion
PCa: prostate cancer
PGSE: pulse gradient spin echo
PSA: prostate specific antigen
PZ: peripheral zone
*R*: cell radius
ROI: region of interest
RPC: reproducibility coefficient
SNR: signal‐to‐noise ratio
*T*_E_: echo time
*T*_R_: repetition time
TZ: transition zone
VASC: vascular
VERDICT: vascular, extracellular and restricted diffusion for cytometry in tumours.

## 1 INTRODUCTION

Prostate cancer (PCa) is the most frequently diagnosed cancer among men in high‐income countries and the second most frequently diagnosed cancer in men worldwide.^1^ Currently, digital rectal examination, serum prostate specific antigen (PSA)—a non‐specific blood test—and trans‐rectal ultrasound‐guided biopsy are the primary diagnostic tools.^2^ Histological analysis remains the main test for PCa diagnosis and grading, and is the standard procedure used to guide treatment. To obtain histological information a portion of tissue is taken from the prostate and observed under a microscope to detect changes in the tissue architecture (biopsy). However, biopsy is invasive and painful, and can result in complications for the patient. Non‐invasive alternatives are highly sought after, as they offer several major benefits: (i) they are safer for the patient; (ii) they do not disrupt tissue, enabling serial examinations and monitoring; (iii) they can examine extended areas of an organ, as opposed to biopsy samples, which are spatially limited. Multi‐parametric prostate MRI is a non‐invasive diagnostic tool, which has been shown to potentially reduce unnecessary primary biopsies in 27% of the patients. However, current non‐invasive imaging techniques lack the sensitivity and specificity required for non‐invasive cancer grading.

Diffusion‐weighted MRI (DW‐MRI) is becoming increasingly important in the assessment of malignant tumours. At the 2008 National Cancer Institute sponsored open consensus conference, experts reached consensus on the use of DW‐MRI as a cancer imaging biomarker.^3^, ^4^ However, current usage of DW‐MRI does not fully exploit its potential. Most cancer DW‐MRI studies use the apparent diffusion coefficient (ADC) for tumour assessment.^5^, ^6^, ^7^, ^8^ ADC reflects the mobility of water molecules within tissue, which provides useful contrast in cancerous tumours, where ADC values are generally observed to be lower than healthy tissue. However, the ADC values in PCa and benign tissue overlap substantially, confounding the overall specificity.^9^, ^10^ ADC is a gross measurement that conflates various physiological parameters, including cell size, shape, permeability, subcellular architecture and vascular perfusion effects.^11^ The dependence of ADC on a variety of histological features simultaneously can mask significant alterations in tissue architecture indicative of malignancy. Moreover, ADC lacks biological specificity and fails to associate contrast changes with particular microstructural effects.^10^

Advanced model‐based imaging techniques can overcome some of the limitations of simplistic diffusion‐based indices, such as ADC, by estimating distinct parameters reflecting separate influences on the signal. In particular, in cancer imaging, VERDICT (vascular, extracellular and restricted diffusion for cytometry in tumours)^12^, ^13^ is a three compartment model‐based DW‐MRI technique that is designed to capture the main microstructural properties of cancerous tissue. VERDICT has shown promising results in preclinical studies for characterizing microstructural tissue properties of xenograft tumour models.^12^ In a clinical setting for PCa, VERDICT has demonstrated the ability to discriminate normal and malignant prostate tissue^13^ and to characterize specific Gleason grades.^14^ However, as with many model‐based techniques,^15^, ^16^, ^17^ VERDICT requires a computationally expensive non‐linear fitting procedure to estimate the model parameters. This prevents immediate inspection of VERDICT parameter maps, which is possible with ADC mapping. Non‐linear fitting is also vulnerable to local minima, which can cause convergence to a sub‐optimal solution, adding to the noise and uncertainty in derived parameter maps. Another limitation of the original VERDICT implementation is that it requires certain parameter constraints for stability, such as fixing the intra‐ and extra‐cellular diffusivities, despite evidence that such parameters may vary.^18^, ^19^

Recently, ultrafast‐fitting algorithms have been developed to address the high computational cost of model‐based microstructure‐imaging techniques.^20^, ^21^, ^22^ Graphical processing units (GPUs) provide a brute‐force solution, using a parallelized approach, to reduce the computational time as in References 20 and 22. Although some overhead lies in GPU‐based design and implementation, once adapted for GPU platforms the fitting time can be reduced by several orders of magnitude compared with standard central processing units, and is limited only by the number of cores on the GPU. Alternatively, through linearization and convex optimization, the accelerated microstructure imaging via convex optimization (AMICO) framework^21^ addresses the cost limitation for each individual fitting operation and is therefore complementary to speed‐ups obtained from GPU computing. AMICO is guaranteed to find the global minimum, albeit of a reformulated objective function, providing more reliable and stable parameter maps. Hence, AMICO potentially affords relaxation of overly restricted constraints, such as fixed diffusivity parameters, that were constrained to stabilize non‐linear fitting. Both solutions have dramatically reduced the computational time of microstructure‐imaging techniques in the brain, e.g. ActiveAx, NODDI and CHARMED.^15^, ^16^, ^23^ Whilst GPUs do not reduce the computational cost, only the computational time, AMICO actually reduces the cost via the linearization. AMICO could be implemented in GPUs and further accelerate the fitting. Rapid‐fitting algorithms are important to analyse the large volume of data arising from studies and clinical trials and, potentially, to improve patient workflow during the clinical process. For example, the UK Biobank imaging project^24^ requires fitting up to 100 000 imaging datasets; only through the use of ultrafast‐fitting algorithms NODDI parameters have been included as imaging phenotypes in the project.^25^, ^26^ Cohorts in PCa studies are also increasingly large^27^, ^28^, ^29^ and thus necessitate similar techniques.

The AMICO framework can be adapted to linearize the estimation of the VERDICT parameters (VERDICT‐AMICO), allowing a fast‐fitting approach.^30^ Here, we present VERDICT‐AMICO and demonstrate its benefits over the original VERDICT implementation. We utilize AMICO to fit the VERDICT model for prostate tissue^13^ to demonstrate reduction in computation time and robust parameter estimation. We explore the reliability of VERDICT‐AMICO using simulated and clinical data. We test whether we can retain the qualitative conspicuity for cancer lesions in parametric maps, and we evaluate the repeatability of the technique within a test–retest experiment. Finally, we use AMICO to demonstrate the possibility of unfixing previously fixed VERDICT parameters.

## 2 THEORY

We first review the original VERDICT model for prostate using non‐linear fitting, and the general AMICO framework.^21^ Then, we describe VERDICT‐AMICO.

### 2.1 VERDICT for prostate

The VERDICT framework characterizes water diffusion in vascular (VASC), extracellular‐extravascular (EES) and intracellular (IC) compartments in tumours.^12^ Mathematically, VERDICT is the sum of three parametric models that describe the DW‐MRI signal in each separate water population assuming zero exchange between them. The normalized diffusion signal for the VERDICT model is

(1) $$S=\sum_{i=1}^{3}f_{i}S_{i}$$

where *f*_*i*_ is the proportion of signal with no diffusion weighting (*b* = 0) from water molecules in population *i* (IC, EES or VASC), 0 ≤ *f*_*i*_ ≤ 1, $\sum_{i=1}^{3}f_{i}=1$. The model has three different volume fraction parameters: *f*_IC_, *f*_EES_ and *f*_VASC_.

For prostate,^13^ the diffusion signal for the IC compartment (*S*_1_) is modelled with impermeable spheres and has *d*_IC_ (IC diffusivity) and *R* (cell radius) as parameters. The sphere (terminology from Reference 17) models particles diffusing inside impermeable spherical boundaries with non‐zero *R* using the Gaussian phase approximation.^31^ In our implementation, as in the previous works that used the sphere model,^12^, ^13^, ^17^ the numerical approximation of the implementation used is 100 roots.

The model for the EES compartment assumes a diffusion tensor (DT) model, in particular an isotropic DT with diffusivity *d*_EES_ as parameter. The normalized DT signal is

(2) $$S_{2}=\exp\left(-bd_{\mathrm{EES}}G^{T}\mathrm{IG}\right)$$

where **I** is the identity tensor, **G** is the gradient direction and *b* = (*Δ* − *δ*/3)(*γδ*|G|)^2^ is the *b*‐value for the pulse gradient spin echo (PGSE) sequence, *Δ* is the time between the onsets of the two pulses, *δ* is the pulse gradient duration and *γ* is the gyromagnetic ratio.

The vascular compartment uses the “astrosticks” model (terminology from Reference 17) for isotropically distributed zero‐diameter restricting cylinders or “sticks”, and has pseudo‐diffusivity *P* as the only parameter. The signal is given by

(3) $$S_{3}=\int S_{r}p\left(n\right)\;\mathrm{dn},R=0,\;S_{r}=\exp\;\left(-\mathrm{bP}\left(n\cdot G^{2}\right)\right)$$

where *n* is the stick direction with uniform distribution *p*, *p*(*n*) = (4*π*)^−1^.

The model has three independent unknown parameters: *f*_IC_, *R* and *f*_EES_. *f*_VASC_ is calculated as *f*_VASC_ = 1 − *f*_IC_ − *f*_EES_, and the diffusion and pseudo‐diffusion coefficients are fixed, as in Reference 13, to *d*_IC_ = *d*_EES_ = 2 × 10^−9^ m^2^/s, *P* = 8 × 10^−9^ m^2^/s.

### 2.2 AMICO

AMICO is a framework that reformulates microstructural imaging techniques as linear systems of equations enabling use of convex optimization techniques (https://github.com/daducci/AMICO/).^21^ More concretely, AMICO uses a dictionary of potential parameter combinations, $\Phi =\left\{\phi_{\mathrm{ij}}\right\}\in {\mathbb{R}}_{+}^{N_{d}\times N_{k}}$, and convex optimization to find the weight vector $\hat{x}\in {\mathbb{R}}_{+}^{N_{k}}\;$for the dictionary elements that best matches the vector containing the *N*_*d*_ measurements $y\in {\mathbb{R}}_{+}^{N_{d}}$. Hence, the system can be formulated as a convex optimization problem as follows:

(4) $$\underset{x\geq 0}{\hat{x}=\text{argmin}}\;\frac{1}{2}\;{\left\|\Phi x-y\right\|}_{2}^{2}+\lambda \Psi \left(x\right)$$

where ‖·‖_2_ is the standard *ℓ*_2_‐norm in *ℝ*^*n*^, the positivity constraint is explicitly imposed as the coefficients *x* correspond to volume fractions, **Ψ**(·) represents a generic regularization function^32^ and the parameter *λ* > 0 controls the trade‐off between data and regularization terms. The AMICO dictionary can also be partitioned into different sub‐matrices to correspond to different compartments considered in the model of choice. We will use this property to adapt AMICO for the three compartments of the VERDICT model. A pre‐defined dictionary for the fitting instead of continuous variables as possible solutions is required to linearize the problem with a fast‐computational performance. Nevertheless, the number of solutions is not limited, as the final parameter estimates are a linear combination of each dictionary value according to the real‐valued weight vector $\hat{x}$.

### 2.3 VERDICT‐AMICO

We adapt the AMICO framework for prostate VERDICT to estimate the three independent unknown parameters as in Reference 13: *f*_IC_, *R* and *f*_EES_.

The dictionary for VERDICT‐AMICO $\Phi =\left\{\phi_{\mathrm{ij}}\right\}\in {\mathbb{R}}_{+}^{N_{d}\times N_{k}}$ is partitioned into three sub‐matrices, corresponding to the VERDICT compartments:

(5) $$\Phi =\left[\Phi^{r}|\Phi^{e}|\Phi^{v}\right]$$

where $\Phi^{r}\in {\mathbb{R}}^{N_{d}\times N_{r}}$, $\Phi^{e}\in {\mathbb{R}}^{N_{d}\times N_{e}}$ and $\Phi^{v}\in {\mathbb{R}}^{N_{d}\times N_{v}}$ each model the IC, EES and vascular contributions of the diffusion signal in the voxel with *N*_*k*_ = *N*_r_ + *N*_e_ + *N*_v_. The regularization function used is the basic Tikhonov regularization with the same *λ* value (*λ* = 0.001) as used in Reference 30.

Here, the full dictionary that mimics the original VERDICT values consists of *N*_*k*_ = 19 entries in total combining three sub‐dictionaries as follows.

- Each column in $\Phi^{r}\in {\mathbb{R}}^{N_{d}\times N_{r}}$ corresponds to the signal attenuation of the water molecules restricted within spheres^17^ with a specific radius. We considered *N*_*r*_ = 17 radii values linearly spaced from 0.01 μm to 15.1 μm. The corresponding signal profiles are estimated according to the “sphere”, assuming an IC diffusion coefficient *d*_IC_ = 2 × 10^−9^ m^2^/s.
- A single compartment, $\Phi^{e}\in {\mathbb{R}}^{N_{d}\times N_{e}}$ where *N*_*e*_ = 1, describes the EES with the same fixed value for the diffusion coefficient *d*_EES_ = 2 × 10^−9^ m^2^/s. The signal model is free isotropic diffusion.
- A single compartment, $\Phi^{v}\in {\mathbb{R}}^{N_{d}\times N_{v}}\;\text{where}\;N_{v}=1,$ is considered to account for the vascular volume fraction. Signal is estimated according to the “astrosticks” model and pseudo‐diffusivity is fixed, *P* = 8 × 10^−9^ m^2^/s.^13^

The estimated coefficients $\hat{x}$ are partitioned into $\left[{\hat{x}}^{r}|{\hat{x}}^{e}|{\hat{x}}^{v}\right]$, and the VERDICT‐AMICO estimated parameters are obtained as

(6) $$f_{\mathrm{IC}}=\sum_{j=1}^{\mathrm{Nr}}{\hat{x}}_{j}^{r}$$

(7) $$R=\frac{\sum_{j=1}^{Nr}R_{j}^{\prime }{\hat{x}}_{j}^{r}}{\sum_{j=1}^{Nr}{\hat{x}}_{j}^{r}}$$

$f_{\mathrm{EES}}={\hat{x}}^{e}$ and $f_{\text{VASC}}={\hat{x}}^{v}$, where *j* ∈ {1, …, *N*_*r*_} indexes the cell radius $R_{j}^{\prime }$ corresponding to the *j*th atom in **Φ**^*r*^. The original VERDICT only assumes one *R* per voxel. When there is more than one *R* per voxel, original VERDICT estimates the average. By averaging the distribution of radii, the two methods are equivalent.

VERDICT‐AMICO inherits parameter constraints from the original non‐linear implementation of the model. The enhanced robustness of the AMICO fitting may be able to avoid these constraints. We hypothesize that VERDICT‐AMICO can estimate a diffusivity parameter without compromising the overall parameter estimation. We previously tested different AMICO dictionaries and observed that different *d*_IC_ values have a small impact on the other parameter estimates with fixed perfusion.^30^ Thus, we unfixed *d*_EES_ and kept *d*_IC_ and *P* fixed with their original values, *d*_IC_ = 2 × 10^−9^m^2^/s and *P*= 8 × 10^−9^m^2^/s.^13^

## 3 METHODS

We first provide details of the patient population, data acquisition and image processing, region of interest (ROI) selection, and synthetic data. We then describe the experiments comparing the two implementations. Finally, we demonstrate the possibility of estimating an additional previously fixed VERDICT parameter.

### 3.1 Patient population

This study has been performed with local ethics committee approval as part as of the INNOVATE clinical trial.^33^ Between April and July 2016, eight men were prospectively recruited and provided informed written consent. The inclusion criteria were the following: (1) suspected PCa or (2) undergoing active surveillance for known PCa. Exclusion criteria were the following: (1) previous hormonal, radiation therapy or surgical treatment for PCa and (2) biopsy within 6 months prior to the scan. Patient characteristics are shown in Table 1.

**Table 1:** ROI characteristics

Patient ID	Patient age [y]	PSA [ng/mL]	ROI ID	Slice (No/14)	No of voxels (Acquisition 2)	Likert (No/5)	Prostate zone	Gleason score (if known)	Biopsy type	ROI group ID
1	63	10.2	1	9	26 (8)	3	PZ	benign	targeted 25/09/2016	1
			2	9	59 (55)	4	PZ	3 + 4		2
			3	9	28 (25)	2	TZ	N/A		3
			4	11	71 (89)	4	TZ	benign		4
2	66	4.7	5	8	10 (10)	2	PZ	N/A	targeted 18/07/2016	5
			6	7	16 (17)	4	PZ	3 + 3		6
			7	7	25 (26)	2	TZ	N/A		3
			8	7	42 (39)	4	TZ	3 + 4		7
3	67	4.86	13	5–6	109 (108)	5	PZ	3 + 4	targeted 31/06/2017	2
			14	8	26 (25)	3	PZ	3 + 3		6
			15	8	28 (26)	2	TZ	N/A		3
			16	8	60 (66)	4	TZ	3 + 3		8
4	73	10.20	9	8	48	2	PZ	benign	targeted 06/10/2016	1
			10	6	29	5	PZ	3 + 4		2
			11	7	16	4	PZ	3 + 4		2
			12	7	27	2	TZ	benign		4
5	49	6.7	17	7	25	2	PZ	benign	TRUS 27/05/2010	1
			18	7	26	2	TZ	benign		4
6	57	4.04	19	8	25	2	PZ	N/A	targeted 04/11/2016.	5
			20	6	29	2	TZ	N/A		3
			21	6	37	4	TZ	3 + 3		8
			22	8	125	2	CZ	N/A		9
7	52	11.1	23	7	26	3	PZ	benign	prostatectomy 02/11/2016	1
			24	6–7	133	5	PZ	4 + 3		10
			25	8	26	2	TZ	3 + 4		7
8	64	6.03	26	11	35	2	PZ	benign	targeted 30/09/2016	1
			27	8	10	4	PZ	benign		1
			28	9	33	2	TZ	benign		4

TRUS, transrectal ultrasound.

### 3.2 Data acquisition and pre‐processing

All patients underwent a standard European Society of Uroradiologists compliant mp‐MRI,^2^ on a 3 T scanner (Achieva, Philips Healthcare, Best, Netherlands) supplemented by VERDICT DW‐MRI. VERDICT DW‐MRI was acquired with PGSE and an optimized imaging protocol for VERDICT prostate adapted from Reference 34 with five *b*‐values of 90–3000 s/mm^2^ in three orthogonal directions using a pelvic coil. Table 2 shows the combinations of *∆*, *δ*,|**G**|, *T*_E_ (echo time) and *T*_R_ (repetition time) for each *b*‐value. For *b* < 100 s/mm^2^ the number of averages (*N*_AV_) = 4 and for *b* > 100 s/mm^2^ *N*_AV_ = 6 with voxel size 1.3 × 1.3 × 5 mm and matrix size 176 × 176. For each *b*‐value (specific *T*_E_‐*T*_R_ combination) a separate *b* = 0 image was acquired to correct for *T*_1_ and *T*_2_ dependence. The scan time for the VERDICT DW‐MRI protocol is 12 min. Three patients (1‐3) were re‐scanned with the VERDICT DW‐MRI sequence without a time interval between the scans.

**Table 2:** Optimized imaging protocol for VERDICT prostate characterization

*b*‐values [s/mm]	*∆* [ms]	*δ* [ms]	|G| [mT/m]	TE [ms]	TR [ms]
90	23.8	3.9	61.2	50	2482
500	31.3	11.4	44.3	65	2482
1500	43.8	23.9	32	90	2482
2000	34.3	14.4	67.7	71	3945
3000	38.8	18.9	60.0	80	3349

*∆*, gradient separation time; *δ*, gradient duration;|G|, gradient strength; TE, echo time; TR, repetition time.

To reduce possible artefacts caused by patient movement during scanning, VERDICT DW‐MRI data was registered using a rigid registration.^35^, ^36^ The transformation matrix was computed using the *b* = 0 images, and then was applied to the *b* = 0 and the subsequent DW‐MRI. We normalize the data using the *b* = 0 images, hence for each voxel the number of normalized measurements is *N*_*d*_= 20 (five *b*‐values with three directions each, and five *b* = 0 images for each *T*_E_). The AMICO dictionary size is limited by the VERDICT DW‐MRI acquisition; we cannot have more dictionary terms than measurements *N*_*d*_ as we are using the simple form of regularization.

### 3.3 ROI selection

**Figure 1:**
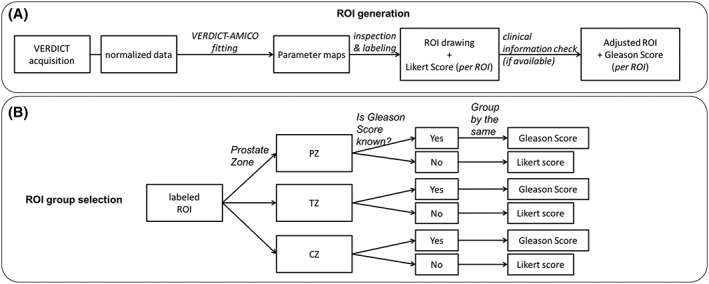
ROI generation and grouping selection. A, Steps followed to generate the individual ROIs. B, Criteria used to group the different ROIs and generate the g‐ROIs

A board‐certified radiologist (EJ) evaluated parametric maps from VERDICT‐AMICO voxelwise fitting (Figure 1A), using Osirix Version 8.0 (Pixmeo, Bernex, Switzerland). The radiologist manually placed ROIs on the VERDICT‐AMICO maps, guided by the conventional mp‐MRI. mp‐MRI findings were reported by an experienced uroradiologist (SP, 11 years of prostate mp‐MRI reporting experience). If biopsy‐confirmed tumours were present, the largest lesion in each zone was manually contoured (transition zone, TZ, peripheral zone, PZ, or central zone, CZ) following the tumour shape. In normal tissue, the ROIs were standard 40 mm^2^ circles. Normal tissue ROIs were drawn in the TZ and the PZ (Likert 2 on mp‐MRI). The Likert score is a five‐point scoring system used for the interpretation of mp‐MRI for PCa.^37^ Clinical biopsy information was also used to confirm where to place the ROIs (Figure 1A).

We grouped the 28 different ROIs according to Gleason score and prostate zone, as we expect them to have similar underlying microstructures. When the Gleason score was unknown we grouped the ROIs according to the Likert score. This procedure resulted in 10 grouped ROIs (g‐ROIs) (Figure 1B). Table 1 provides details for the ROIs and g‐ROIs. We compute the signal‐to‐noise ratio (SNR) in the VERDICT data using the method of Dikaios et al.,^38^ which gives a mean SNR of 19.97 (range 10.56 to 40.38).

### 3.4 Synthetic data

We generate synthetic data with 30 different values for *f*_IC_, *f*_EES_ and *R* (equally spaced from 0.02 μm to 15 μm) creating all the possible parameter combinations that satisfy 0 ≤ *f*_IC_ + *f*_EES_ ≤ 1. We only set values of *R* for *f*_IC_ > 0. The generated signal accounts for Rician noise,^39^ with *b* = 0 SNR set to 20, 50, ∞. We include 10 different noise instances for each SNR (20 and 50), which are sufficient to guarantee reproducible estimates. The error of our estimated signal plateaus after five instances for SNR = 20 and two instances for SNR = 50. Thus, for each instance of noise we had a total of 13 050 voxels.

To generate the synthetic data and for the original fitting implementation we used the open‐source Camino toolkit.^40^ Camino uses an iterative optimization procedure with the Levenberg–Marquardt algorithm that mitigates the problem of local minima and Rician noise, as in References 13 and 17. All experiments were conducted on a 3.1 GHz Intel Core i7, 8 GB RAM DDR3.

### 3.5 Fitting performance experiments

We test the VERDICT model using both the original non‐linear fitting and the linear VERDICT‐AMICO. We use synthetic data to compare both implementations for accuracy against ground‐truth estimates. Then, to test the fitting performance with clinical data, we compare the variance of the estimated parameters in different known tissue types. We analyse ROIs within the same g‐ROIs together. We then contrast the parametric maps in terms of run‐time and fitting performance.

Two board‐certified radiologists (SP and EJ) examined the VERDICT‐AMICO parametric maps for qualitative tumour conspicuity (contrast between tumour and surrounding tissue) to ensure tumour enhancement and clinical relevance. Then, we compared the chi‐squared objective function maps (*f*_obj_), which are sum of square differences adjusted to account for offset Gaussian noise, to evaluate the robustness of the fitting, as in Reference 17. Finally, we test the repeatability of both procedures in three patients (scan‐rescan) using Bland–Altman agreement analysis.^41^ We emphasize that the different parameter maps for both VERDICT and VERDICT‐AMICO should be viewed side by side to aid in their interpretation. For example, the estimated *R* value is less relevant if the *f*_IC_ is almost zero in that region, and in areas where *f*_obj_ is high all estimated parameters are less reliable.

### 3.6 *d*_EES_ estimation with VERDICT‐AMICO experiment

Here, we use both fitting methods (original non‐linear and VERDICT‐AMICO) to estimate the extra parameter (*d*_EES_) in two datasets, and we examine the run‐time and goodness of fit. The VERDICT‐AMICO dictionary with unfixed *d*_EES_ is

- *N*_*r*_ = 13 different radii (linearly spaced from 0.01 μm to 15.1 μm) with fixed *d*_IC_ = 2 × 10^−9^ m^2^/s.
- *N*_*e*_ = 5 diffusion coefficients for EES: *d*_EES_ = 1.1 × 10^−9^, 1.6 × 10^−9^, 2.1 × 10^−9^, 2.6 × 10^−9^ and 3.1 × 10^−9^ m^2^/s.
- *N*_*v*_ = 1, *P* = 8 × 10^−9^ m^2^/s.

Details about the rationale and the discretization of *d*_EES_ can be found in Reference 30.

## 4 RESULTS

### 4.1 Synthetic data

**Figure 2:**
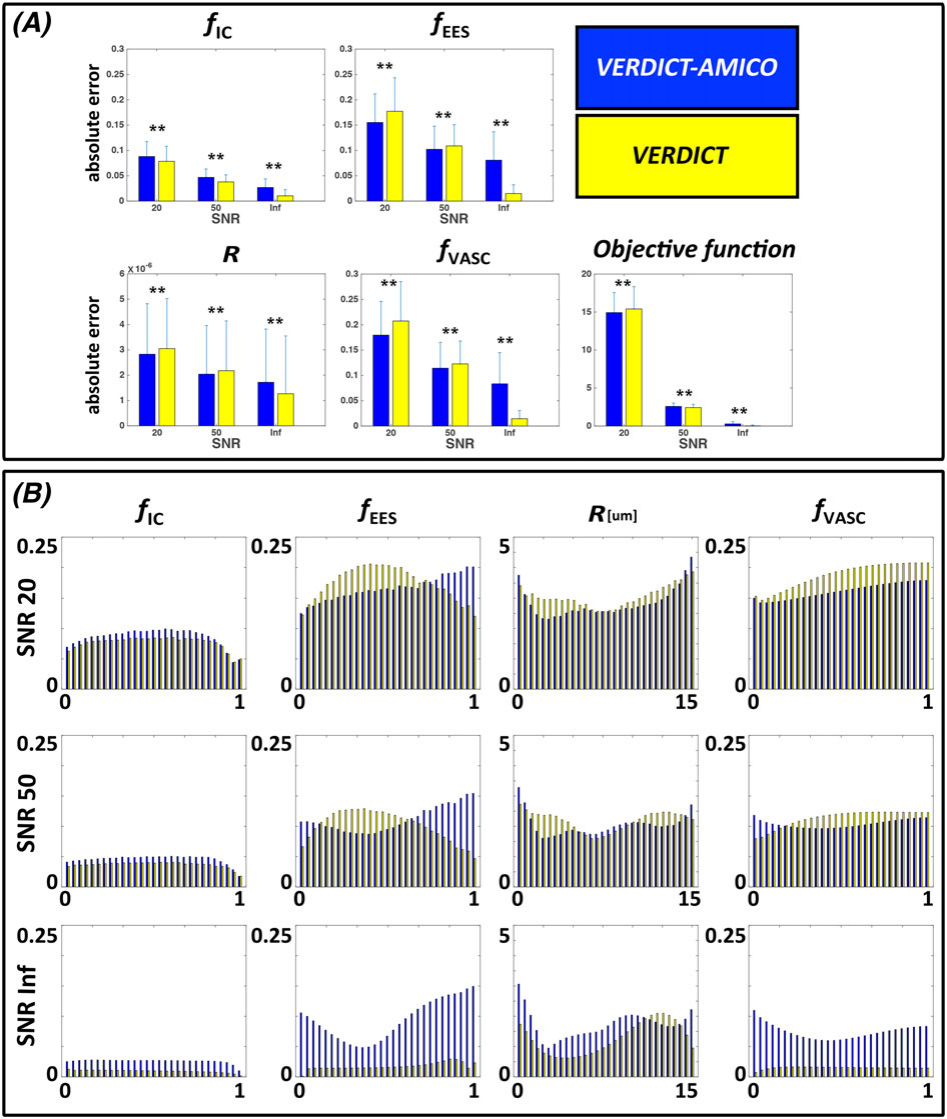
Quantitative evaluation using synthetic data. Performance comparison of the two fitting implementations of VERDICT as a function of SNR for the synthetic data. A, Mean absolute error obtained with both models with respect to the ground‐truth. Bars are the standard deviations of the errors. B, Absolute errors for each estimated parameter as a function of original synthetic values. *f*_IC_, intracellular volume fraction; *f*_EES_, extracellular‐extravascular volume fraction; *R*, radius [μm]; *f*_VASC_, vascular volume fraction

Figure 2 shows the performance of both fitting methods, VERDICT and VERDICT‐AMICO, using synthetic data as a function of SNR. Figure 2A shows the absolute errors for each estimated parameter. We compute the mean absolute errors for the overall combination of the input parameters. The mean error reduces as SNR increases for both procedures. However, this reduction is less obvious for VERDICT‐AMICO, mainly because it performs poorly at infinite SNR. Nevertheless, at a typical noise level for clinical acquisitions (SNR = 20), VERDICT‐AMICO generally exhibits a lower absolute error than VERDICT (*p* < 0.001 for the objective function in the absolute error comparison). VERDICT‐AMICO performs worse for *f*_IC_, but the overall fitting, as revealed by the objective function errors, is better. VERDICT‐AMICO maintains estimated fitting precision comparable to VERDICT when the SNR is reduced. The reductions in the fitting precision estimates measured by the ratio between the standard deviation at SNR = 20 and SNR = ∞ for *f*_IC_, *f*_EES_, *f*_VASC_ and *R* are 1.75, 1.00, 1.08 and 0.95 for VERDICT‐AMICO, and 2.39, 3.84, 4.82 and 0.87 for VERDICT.

Figure 2B presents the mean absolute errors for each estimated parameter as a function of the ground‐truth synthetic values to highlight the dependence of fitting errors. The estimated parameters *f*_IC_ and *R* at SNR = 20 and SNR = 50 show approximately constant error scores across the ground‐truth values for both fitting procedures. For *f*_IC_, the AMICO fitting procedure makes slightly larger errors than the original fitting. In contrast, the VERDICT‐AMICO error in *f*_EES_ increases with the real *f*_EES_ value, and the original fitting for VERDICT is worse when *f*_EES_ < 0.75 at SNR = 20 or 0.17 < *f*_EES_ < 0.58 at SNR = 50.

### 4.2 Clinical data

We also evaluate the performance of both methods using clinical data. First, we compare the computational times for parametric maps (Table 3). The VERDICT‐AMICO formulation reduces the processing time by more than three orders of magnitude, from 6.55 s/voxel to 1.78 ms/voxel. To explore the acceleration provided by AMICO, independent of the programming language used, we calculated the processing time for VERDICT (Camino) using a full MATLAB pipeline (instead of Java) in 600 voxels. The mean processing time for VERDICT using the MATLAB pipeline is 0.75 s/voxel, still two orders of magnitude slower than VERDICT‐AMICO.

**Table 3:** Time required to compute each prostate ROI with non‐linear (VERDICT) and linear (VERDICT‐AMICO) fitting procedures

Patient ID	Slice (No/14)	Original VERDICT [h]	VERDICT‐AMICO [s]	No of voxels	VERDICT/voxel [s]	VERDICT‐AMICO/voxel [ms]
1 acq 1	9	2.319	2.824	1240	0.644	2.278
	11	2.200	1.8052	1118	7.087	1.615
1 acq 2	9	2.392	2.33	1220	7.060	1.915
	11	2.120	1.787	1098	6.952	1.628
2 acq 1	8	1.86	1.652	1038	6.456	1.592
	7	1.889	1.752	1064	6.393	1.647
2 acq 2	8	1.823	1.745	1038	6.323	1.682
	7	1.926	1.963	1064	6.517	1.845
3 acq 1	5–6	3.182	2.254	1692	6.771	1.332
	8	2.097	2.236	1062	7.109	2.106
3 acq 2	5–6	3.079	2.148	1609	6.889	1.335
	8	2.150	1.989	1086	7.130	1.832
4	8	1.748	1.788	840	7.493	2.129
	6	1.610	1.835	809	7.165	2.269
	7	1.931	1.694	956	7.275	1.772
5	7	1.998	1.708	1105	6.512	1.546
6	8	1.751	1.700	987	6.388	1.723
	6	1.653	1.446	951	6.259	1.521
7	7	0.994	1.175	528	6.783	2.227
	6–7	1.910	1.546	1021	6.737	1.515
	8	1.232	1.307	605	7.333	2.161
8	11	1.892	1.529	1028	6.629	1.488
	8	1.211	1.213	652	6.689	1.861
	9	1.412	1.343	773	6.578	1.738
Mean					6.548 s/voxel	1.781 ms/voxel

**Figure 3:**
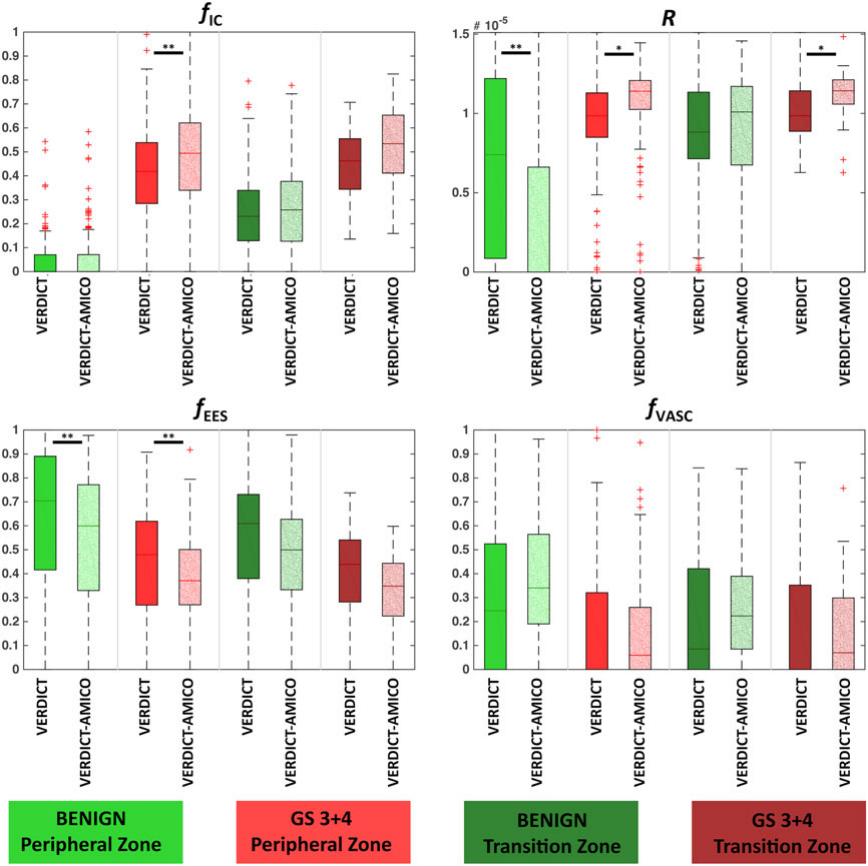
Quantitative comparison of VERDICT maps versus VERDICT‐AMICO. Parameter estimates for all voxels within the ROIs of four different g‐ROIs (1, 2, 4 and 7) for all the estimation procedures (VERDICT and VERDICT‐AMICO). *f*_IC_, intracellular volume fraction; *f*_EES_, extracellular‐extravascular volume fraction; *R*, radius [μm]; *f*_VASC_, vascular volume fraction. GS, Gleason score. Arbitrary units. *p < 0.01; ** *p* < 0.001

We then compare parameter estimates for all voxels within ROIs. Figure 3 illustrates the parameter estimates for all voxels within four g‐ROIs with a confirmed tissue type (normal and Gleason Score 3 + 4 in TZ and PZ). VERDICT‐AMICO shows similar estimates to VERDICT for *f*_IC_ and *f*_EES_, with Pearson correlations *r* ^2^ (sum of squared error) of 0.91 (0.049) and 0.74 (0.079), respectively. The VERDICT‐AMICO estimates for *f*_IC_ and *R* in tumour lesions are statistically significantly higher, and *f*_EES_ values are lower compared with VERDICT, but both methods produce physiologically plausible values within the same range. The precisions of the fit estimates (*σ*) are better for the AMICO implementation, being 0.15, 0.16, 0.20 and 0.33 × 10^−5^ for *f*_IC_, *f*_EES_, *f*_VASC_ and *R* respectively, compared with 0.15, 0.24, 0.24 and 0.41 × 10^−5^ for the original implementation. Individual ROI analysis yields similar results (not shown).

**Figure 4:**
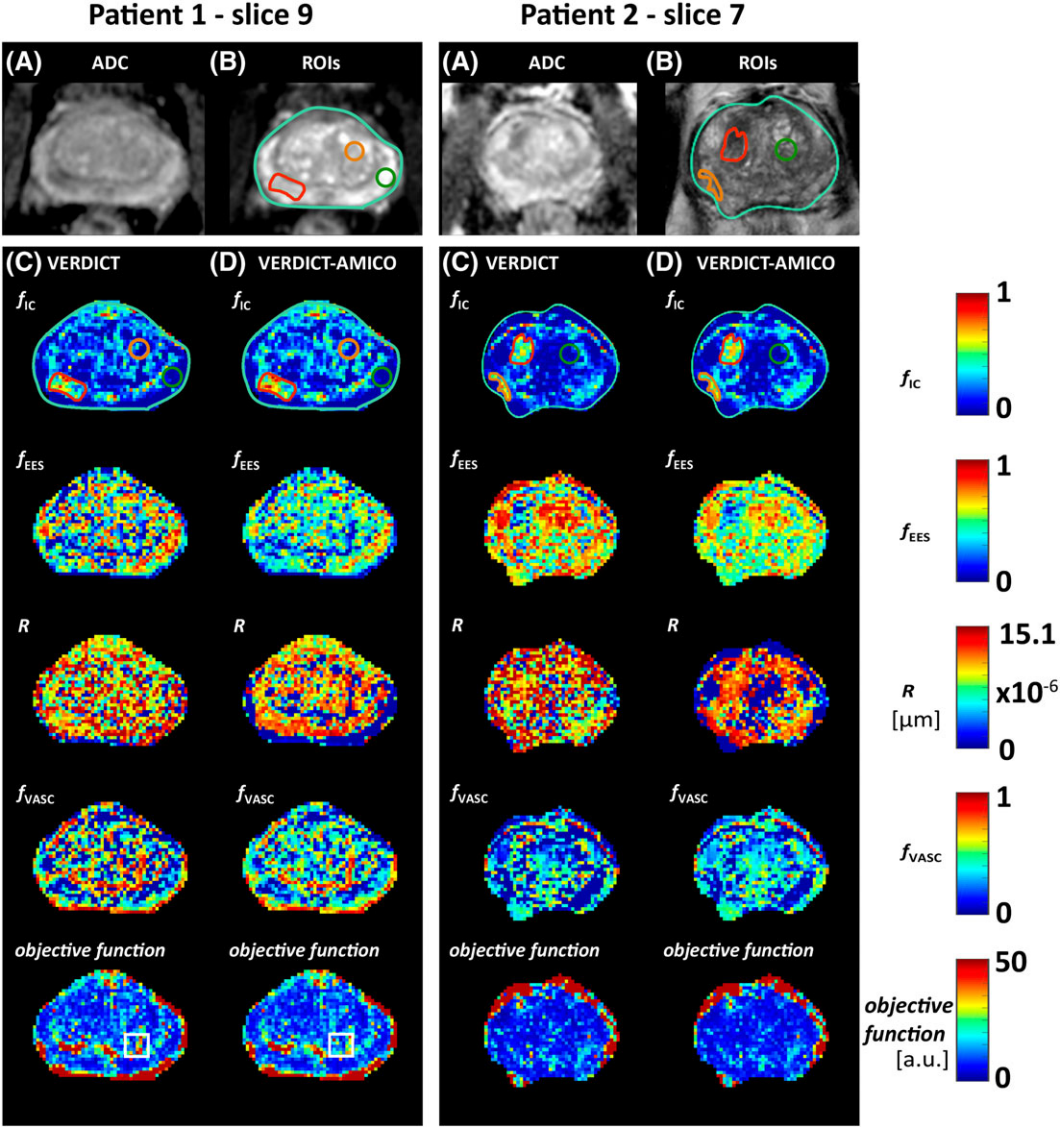
Qualitative comparison of VERDICT maps for two different patients: VERDICT versus VERDICT‐AMICO. A, Standard ADC image. B, Corresponding ROIs on a *T*_2_ image: the whole prostate (light green) and ROIs 1 and 7 (green), 3 and 6 (orange) and 2 and 8 (red). ROI description shown in Table 1. C, D, Parametric maps for VERDICT and VERDICT‐AMICO, respectively. ROIs are overdrawn on the *f*_IC_ map for illustrative purposes. The white square highlights a region with greater homogeneity in the objective function for VERDICT‐AMICO compared with VERDICT. *f*_IC_, intracellular volume fraction; *f*_EES_, extracellular‐extravascular volume fraction; *R*, radius [μm]; *f*_VASC_, vascular volume fraction. a.u., arbitrary units

Figure 4 presents a qualitative comparison of parametric maps for two patients with a standard ADC image from the mp‐MRI (A), the corresponding ROIs for that slice superimposed onto a *T*_2_ reference image (B) and VERDICT (C) and VERDICT‐AMICO (D) maps. The two board‐certified radiologists agreed that tumour conspicuities are similar for the two fitting methods and reveal similar qualitative differences between tumour and normal tissue. For example, in both cases *f*_IC_ is higher and *f*_EES_ is lower in tumour compared with normal tissue, as seen in References 13 and 42. In general, the two implementations present similar behaviours. Maps obtained using AMICO appear less noisy than those with the original non‐linear fitting. This effect is more evident for the radius map.

**Figure 5:**
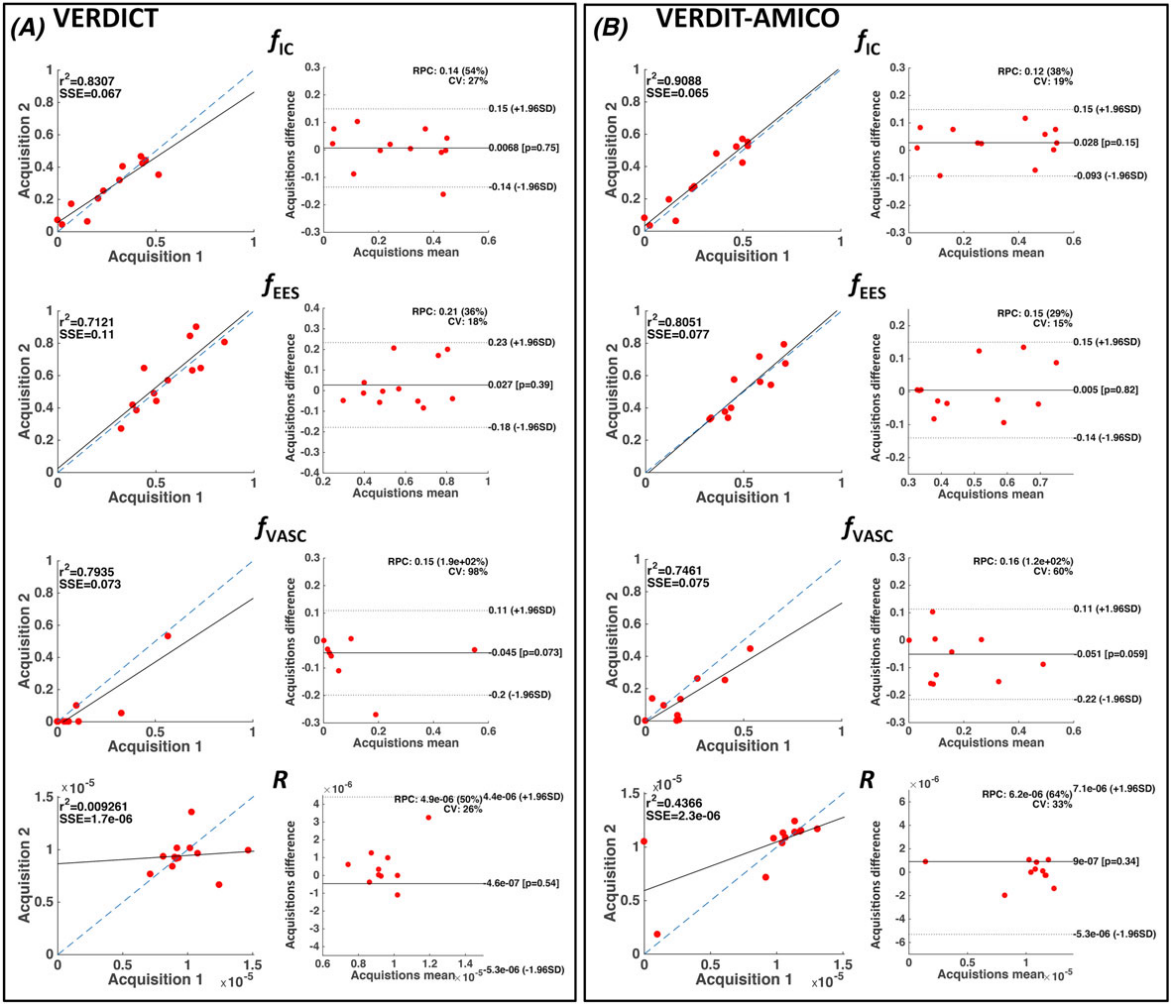
Repeatability. Correlation plots for parameter estimation comparison between two different acquisitions for the 12 repeated ROIs (ROIs 1 to 12) for VERDICT (A) and VERDICT‐AMICO (B). Median values for all voxels within each ROI were used. *f*_IC_, intracellular volume fraction; *f*_EES_, extracellular‐extravascular volume fraction; *R*, radius [μm]; *f*_VASC_, vascular volume fraction; *r* ^2^, Pearson's *R*‐value squared; SSE, sum of squared error; SD, standard deviation

Figure 5 presents correlations of the estimated parameters from the repeatability experiment for 12 ROIs with both methods. Overall, most parameters are repeatable with high correlation coefficients. The AMICO implementation has the highest correlation coefficients (except for *f*_VASC_). For both methods, *R* appears the least repeatable. We further test the repeatability of *R* by recalculating correlation coefficients whilst excluding ROIs with fewer than 10 voxels (ROI 1 from Table 1), and voxels where *f*_IC_ was too small to produce a reliable signal (*f*_IC_ < 0.001). The repeatability does not improve considerably for VERDICT‐AMICO (*r*^2^ = 0.037, SSE = 1.5 × 10^−6^) or for the non‐linear fitting (*r*^2^ = 0.034, SSE = 2.1 × 10^−6^). However, the reproducibility coefficient (RPC) and the coefficient of variation (CV) from the Bland–Altman test are better for VERDICT‐AMICO (RPC = 29%, CV = 15%) than for VERDICT (RPC = 53%, CV = 27%).

### 4.3 *d*_EES_ estimation with VERDICT‐AMICO

**Figure 6:**
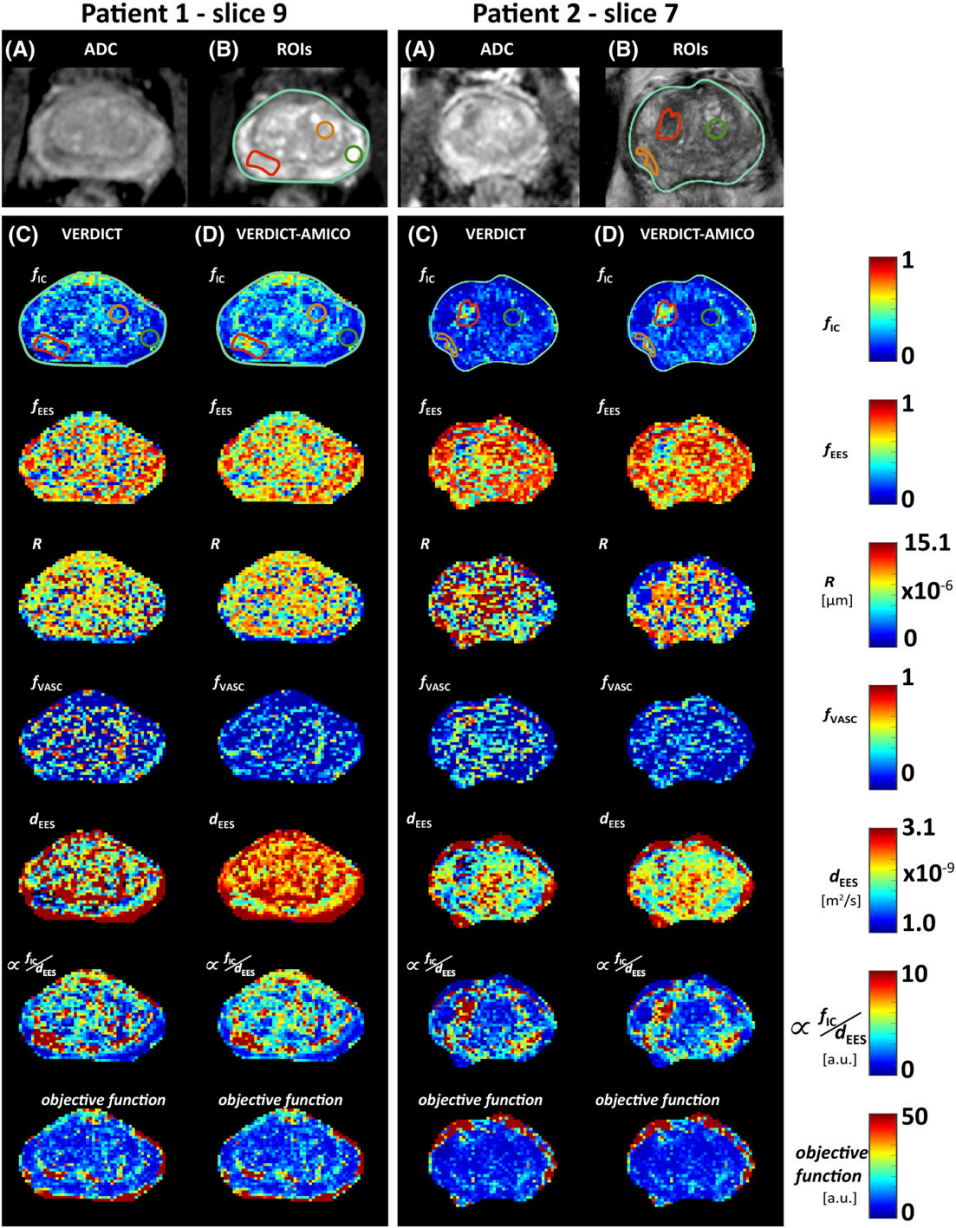
Qualitative comparison of VERDICT maps after unfixing *d*_EES_ for two different patients: VERDICT versus VERDICT‐AMICO. A, Standard ADC image. B, Corresponding ROIs on a *T*_2_ image: the whole prostate (light green) and ROIs 1 and 7 (green), 3 and 6 (orange) and 2 and 8 (red). ROI description shown in Table 1. C, D, Parametric maps for VERDICT and VERDICT‐AMICO after unfixing *d*_EES_, respectively. ROIs are overdrawn on the *f*_IC_ map for illustrative purposes. *f*_IC_, intracellular volume fraction; *f*_EES_, extracellular‐extravascular volume fraction; *R*, radius [μm]; *f*_VASC_, vascular volume fraction; *d*_EES_, extracellular‐extravascular diffusivity [m^2^/s]. a.u., arbitrary units

Table 4 shows the computational time for both fitting implementations after unfixing *d*_EES_. In both cases, the extra parameter requires more computational time, 2.20 ms/voxel and 7.68 s/voxel for the linear and the non‐linear fitting respectively. Figure 6 demonstrates a visual comparison of the maps for both fittings. Note the additional maps: the *d*_EES_ map, and an example of a parametric combination (*f*_IC_/*d*_EES_) that exhibits increased tumour conspicuity. Maps reveal reduced *d*_EES_ and *f*_EES_ in lesions while retaining a high *f*_IC_. We also observe that *f*_VASC_ is lower than with the original model. VERDICT‐AMICO with the new dictionary achieves lower objective function values than with fixed *d*_EES_.

**Table 4:** Time required to compute each prostate ROI after unfixing *d*_EES_ for two different patients with non‐linear (VERDICT) and linear (VERDICT‐AMICO) fitting procedures

Patient ID	Slice (No/14)	Free *d*_EES_ VERDICT [h]	Free *d*_EES_ VERDICT‐AMICO [s]	No of voxels	Free *d*_EES_ VERDICT/voxel [s]	Free *d*_EES_ VERDICT‐AMICO/voxel [ms]
1 acq 1	9	2.72	3.23	1240	7.90	2.60
	11	2.49	2.50	1118	8.03	2.23
1 acq 2	9	2.68	2.68	1220	7.91	2.20
	11	2.44	2.26	1098	8.02	2.06
2 acq 1	8	2.13	2.16	1038	7.39	2.08
	7	2.15	2.30	1064	7.30	2.17
2 acq 2	8	2.16	2.19	1038	7.49	2.11
	7	2.18	2.22	1064	7.38	2.09
Mean					7.68 s/voxel	2.19ms/voxel

## 5 DISCUSSION

In this study, we adapted the AMICO framework for the VERDICT technique for PCa characterization to reduce the computational cost of the original fitting. We tested VERDICT‐AMICO against the non‐linear fitting procedure in terms of (a) accuracy against ground‐truth simulation values, (b) computational time, (c) fitting precision in different tissue types, (d) qualitative tumour conspicuity and (e) repeatability. Finally, we demonstrated the estimation of an extra VERDICT parameter: the EES diffusivity (*d*_EES_). The principal benefit of the AMICO framework in prostate is that it provides an acceleration factor of several orders of magnitude compared with non‐linear fitting, whilst offering robust parameter estimation, and without significantly affecting the parameter maps.

First, we tested the accuracy of both fitting procedures using synthetic data with an extensive range of parameter values within biophysical limits (Figure 2). We also checked the accuracy at different SNR levels. For finite SNR, VERDICT‐AMICO appears to produce errors that vary less, as a function of parameter values, than the original VERDICT. For infinite SNR, VERDICT‐AMICO performance is worse than that of VERDICT; this is an artefact of the regularization term—regularization parameters fail when dealing with perfect data because numerical calculations may cause instabilities and yield an unsatisfactory solution.^43^ However, with an SNR of 20, similar to the level in our clinical samples, VERDICT‐AMICO is more accurate than VERDICT for most parameters. In the future, to improve the fitting procedure, we could adjust the regularization term ad hoc by estimating the SNR for each region. However, this solution requires a prostate segmentation as input, since the SNR is different in different image regions. The noise model may also influence our results, as AMICO assumes Gaussian noise. Many papers suggest that when the SNR is high enough the noise can be approximated as Gaussian.^39^ For SNR = 20, AMICO with Gaussian noise has acceptable results; however, future versions of AMICO will incorporate non‐Gaussian noise.

Second, we examined the computational run‐time for the two fitting procedures. Results in Table 3 illustrate that AMICO provides an acceleration factor of several orders of magnitude compared with the non‐linear fitting, while the parameter estimation remains similar to the original VERDICT fitting. This speed‐up makes it practical for researchers to analyse entire volumes, rather than single slices, of prostate data using standard computers. AMICO thus allows the computation of parametric maps without human intervention for precise gland/lesion segmentation. Additionally, AMICO would allow VERDICT to be computed directly during the scan, similar to the current use of ADC in clinics. Analysing the whole prostate enables the observation of multiple lesions from large datasets, facilitating translation of the method to routine clinical use. VERDICT‐AMICO can fit the whole volume of interest (30 976 voxels) in less than 1 minute. Improvement in computation time could also potentially be achieved using large GPUs.^20^, ^22^ However, AMICO provides an entirely complementary and direct reduction in computational cost.

We measured the parameter precision in various prostate regions in benign and cancerous tissue (Figure 3). We observed differences in *f*_IC_ among normal PZ and TZ, with higher *f*_IC_ for normal TZ. This could potentially be due to the fact that TZ is the main site of origin of prostatic hyperplasia, which is also characterized by a higher volume of epithelial cells, much like tumours.^44^ Like VERDICT, VERDICT‐AMICO tends to overestimate vascular fraction; we see higher *f*_VASC_ in the presented results than we would expect from histology.^44^ This could be a similar effect to the overestimation of cerebrospinal fluid volume fraction in white matter with NODDI that also arises as a result of fixing diffusivity parameters.^45^ We no longer observe vasculature overestimation after unfixing the EES diffusivity.

Visual comparison (Figure 4) by board‐certified radiologists showed that the two methods produce similar parametric maps. VERDICT‐AMICO retains the qualitative conspicuity in lesions and revealed the same trends observed in Figure 3. Overall, the VERDICT‐AMICO formulation produces smoother and more robust maps suitable for radiological inspection, as it enables location of the global minimum more reliably than the original fitting. Greater homogeneity in the minimum objective function maps support this assertion (see the highlighted white square in Figure 4).

We further tested the clinical applicability of VERDICT‐AMICO by investigating the repeatability of the parameter estimates (Figure 5). Results showed high levels of repeatability for most parameters for both methods. However, the AMICO implementation improves repeatability over the non‐linear fitting for *f*_IC_ and *f*_EES_. For *R*, the repeatability is low for both methods. Future work will investigate this result using histology. In our dataset, parameter estimation in tumour regions was less repeatable than in normal tissue (results not shown). This is probably because tumour tissue can be highly heterogeneous, especially when compared with normal tissue, so small errors in the ROI definition can produce greater differences. A more extensive dataset with many tumour types is needed to further test parameter repeatability in cancer lesions.

Next, we examined whether the VERDICT‐AMICO improvement in computational time was retained when estimating an additional VERDICT parameter, the diffusivity *d*_EES_.^30^ We also estimated *d*_EES_ with the original non‐linear fitting (Figure 6). As expected, the parametric maps presented similar results for both methods. VERDICT‐AMICO exhibits the same improvement over the original fitting for computational time and robustness after unfixing *d*_EES_. For both fittings, the parameter estimates with the new dictionary are more biophysically plausible and in closer agreement with histology^44^ than the original VERDICT estimates (fixed *d*_EES_). This is especially evident for the vascular volume fraction *f*_VASC_. This is an important improvement, as the general lower estimation of vasculature is in line with histological analysis of our samples (not shown), showing a vascular component of approximately 1%. Unfixing *d*_EES_ could allow our model to account better for the lumen space as a separate component from stroma; tissue previously captured as part of the vascular compartment might be now part of the EES compartment. A very small vascular component could explain the noisier *f*_VASC_ estimates compared with the rest of the parameters. However, we note that our samples lack highly vascularized cancer lesions. Currently, the literature relating vascularization and aggressive cancers is controversial for prostate.^46^, ^47^, ^48^, ^49^, ^50^ However, there is general agreement that increased angiogenesis is an important factor in determining tumour development and prognosis.^51^ Aggressive cancers need to be quickly identified and they do show significant vasculature; therefore, it is important to capture the vasculature component of the tissue, which justifies the inclusion of a vascular component in our model.

In Figure 6 we compare VERDICT‐AMICO and VERDICT with an extra unfixed parameter, *d*_EES_. As in the original VERDICT‐AMICO implementation with fixed *d*_EES_, the rest of the estimated parameters (*f*_IC_, *R*, *f*_EES_) do not generally hit the upper boundary constraint compared with the original VERDICT implementation. In Figure 6, *R* hits the upper boundary in some voxels for VERDICT also after unfixing *d*_EES_, which evidences that AMICO improves and stabilizes the fitting. Despite the more realistic estimates after unfixing *d*_EES_, both VERDICT and VERDICT‐AMICO lose some tumour conspicuity in the *f*_IC_ maps compared with the original model, which is a clinically important aspect. However, the contrast remains among the full set of maps. For example, we show that the conspicuity can be recovered and perhaps enhanced using the *f*_IC_/*d*_EES_ combination. One possible explanation for the enhanced conspicuity could be a decreased inter‐cell space and increased tortuosity, which is consistent with a higher partial volume of epithelial cells and loss of lumen space.^11^ VERDICT with fixed *d*_EES_ may still have clinical utility for highlighting tumours. In the future, we will run a comprehensive study involving clinicians to further compare the potential clinical use of the two implementations.

The main limitation of the AMICO framework is that the fitting results depend to some extent on the dictionary values, as the regularization has to be set empirically.^21^ For this reason, we used histological information to guide the selection of dictionary values. Biopsy results confirm that the estimated maps provide plausible values even with the limited dictionary. However, more samples from larger datasets are required to validate the VERDICT parameters with histology, and this is beyond the scope of this study. Future efforts with larger cohorts will focus on clinical VERDICT parameter validation, using methods such as those of Reference 52 to further investigate VERDICT as a non‐invasive MRI‐based cancer biomarker. VERDICT‐AMICO also inherits the limitations and assumptions of the VERDICT prostate model, which need to be taken into consideration when interpreting the parameter estimates. For example, the current model does not account for permeability between the different components or for large differences in *T*_2_ within the same voxel (*T*_2_ heterogeneity),^53^ which could cause bias in the parameter estimation. In the future, we will study the *T*_2_ and permeability effects and incorporate the findings into the VERDICT model. Nevertheless, the results from this study and previous work provide good evidence that VERDICT‐AMICO can provide useful information, improving current methods.

To conclude, VERDICT‐AMICO's fast and robust fitting highlights important microstructural differences between tumours and normal prostate tissue in seconds, which is crucial for utilizing VERDICT as a diagnostic tool. Longer‐term, fast‐fitting algorithms such as AMICO will be essential for the translation of promising techniques like VERDICT to widespread clinical applications. Our results suggest that VERDICT‐AMICO maps provide additional value over the original VERDICT prostate implementation, and we hope they will be a valuable source of clinical imaging biomarkers. However, further studies are required to validate this assumption. The VERDICT‐AMICO implementation will be freely available online (http://mig.cs.ucl.ac.uk Resources tab). VERDICT‐AMICO shows promise as a MRI‐based tool for PCa grading and detection, which could be used to enhance the diagnostic potential of current prostate mp‐MRI.

## References

[1] Torre LA , Bray F , Siegel RL , Ferlay J , Lortet‐Tieulent J , Jemal A . Global Cancer Statistics, 2012. CA Cancer J Clin. 2015;65(2):87‐108. 10.3322/caac.21262 25651787

[2] Barentsz JO , Richenberg J , Clements R , et al. ESUR prostate MR guidelines 2012. Eur Radiol. 2012;22(4):746‐757. 10.1007/s00330-011-2377-y 22322308 PMC3297750

[3] Padhani AR , Liu G , Koh DM , et al. Diffusion‐weighted magnetic resonance imaging as a cancer biomarker: consensus and recommendations. Neoplasia 2009;11(2):102–125. http://www.pubmedcentral.nih.gov/articlerender.fcgi?artid=2631136&tool=pmcentrez&rendertype=abstract. Accessed August 31, 2015. 19186405 10.1593/neo.81328 PMC2631136

[4] Prostate Cancer: diagnosis and management. *Clinical Guideline [CG175]* NICE. http://www.nice.org.uk/guidance/CG175 Accessed October 16, 2017.

[5] Giles SL , Morgan VA , Riches SF , Thomas K , Parker C , deSouza NM . Apparent diffusion coefficient as a predictive biomarker of prostate cancer progression: value of fast and slow diffusion components. Am J Roentgenol. 2011;196(3):586‐591. 10.2214/AJR.10.5016 21343500

[6] Peng Y , Jiang Y , Antic T , et al. Apparent diffusion coefficient for prostate cancer imaging: impact of b values. Am J Roentgenol. 2014;202(3):W247‐W253. 10.2214/AJR.13.10917 24555621

[7] Squillaci E , Manenti G , Cova M , et al. Correlation of diffusion‐weighted MR imaging with cellularity of renal tumours. Anticancer Res. 2004;24(6):4175‐4179. 10.1259/bjr/26525081. 15736469

[8] Afaq A , Andreou A , Koh DM . Diffusion‐weighted magnetic resonance imaging for tumour response assessment: why, when and how? Cancer Imaging. 2010;10:S179‐S188. 10.1102/1470-7330.2010.9032. 20880779 PMC2967137

[9] Gupta RT , Kauffman CR , Garcia‐Reyes K , et al. Apparent diffusion coefficient values of the benign central zone of the prostate: comparison with low‐ and high‐grade prostate cancer. Am J Roentgenol. 2015;205(2):331‐336. 10.2214/AJR.14.14221 26204283 PMC4807133

[10] Bourne R , Panagiotaki E . Limitations and prospects for diffusion‐weighted MRI of the prostate. Diagnostics. 2016;6(2):21 10.3390/diagnostics6020021 PMC4931416 27240408

[11] Chatterjee A , Watson G , Myint E , Sved P , McEntee M , Bourne R . Changes in epithelium, stroma, and lumen space correlate more strongly with Gleason pattern and are stronger predictors of prostate ADC changes than cellularity metrics. Radiology. 2015;277(3):751‐762. 10.1148/radiol.2015142414 26110669

[12] Panagiotaki E , Walker‐Samuel S , Siow B , et al. Noninvasive quantification of solid tumor microstructure using VERDICT MRI. Cancer Res. 2014;74(7):1902‐1912. 10.1158/0008-5472.CAN-13-2511 24491802

[13] Panagiotaki E , Chan RW , Dikaios N , et al. Microstructural characterization of normal and malignant human prostate tissue with vascular, extracellular, and restricted diffusion for cytometry in tumours magnetic resonance imaging. Invest Radiol. 2015;50(4):218‐227. 10.1097/RLI.0000000000000115 25426656

[14] Brizmohun M , Johnston E , Latifoltojar A , et al. The intracellular component of VERDICT (Vascular, Extracellular, and Restricted Diffusion for Cytometry in Tumors) MRI distinguishes Gleason 4 pattern better than apparent diffusion coefficient. Paper presented at: Joint Annual Meeting ISMRM‐ESMRMB 2018; Paris, France. http://discovery.ucl.ac.uk/10047404/. Accessed August 2, 2018.

[15] Alexander DC , Hubbard PL , Hall MG , et al. Orientationally invariant indices of axon diameter and density from diffusion MRI. Neuroimage. 2010;52(4):1374‐1389. 10.1016/j.neuroimage.2010.05.043 20580932

[16] Zhang H , Schneider T , Wheeler‐Kingshott CA , Alexander DC . NODDI: practical in vivo neurite orientation dispersion and density imaging of the human brain. Neuroimage. 2012;61(4):1000‐1016. 10.1016/j.neuroimage.2012.03.072 22484410

[17] Panagiotaki E , Schneider T , Siow B , Hall MG , Lythgoe MF , Alexander DC . Compartment models of the diffusion MR signal in brain white matter: a taxonomy and comparison. Neuroimage. 2012;59(3):2241‐2254. 10.1016/j.neuroimage.2011.09.081 22001791

[18] Kaden E , Kruggel F , Alexander DC . Quantitative mapping of the per‐axon diffusion coefficients in brain white matter. Magn Reson Med. 2016;75(4):1752‐1763. 10.1002/mrm.25734 25974332 PMC4975722

[19] Jelescu IO , Veraart J , Fieremans E , Novikov DS . Degeneracy in model parameter estimation for multi‐compartmental diffusion in neuronal tissue. NMR Biomed. 2016;29(1):33‐47. 10.1002/nbm.3450 26615981 PMC4920129

[20] Hernández M , Guerrero GD , Cecilia JM , et al. Accelerating fibre orientation estimation from diffusion weighted magnetic resonance imaging using GPUs. PLoS ONE. 2013;8(4):e61892 10.1371/journal.pone.0061892 23658616 PMC3643787

[21] Daducci A , Canales‐Rodríguez EJ , Zhang H , Dyrby TB , Alexander DC , Thiran J‐P . Accelerated Microstructure Imaging via Convex Optimization (AMICO) from diffusion MRI data. Neuroimage. 2015;105:32‐44. 10.1016/j.neuroimage.2014.10.026 25462697

[22] Harms R , Fritz F , Tobisch A , Goebel R , Roebroeck A . Robust and fast nonlinear optimization of diffusion MRI microstructure models. Neuroimage. 2017;155:82‐96. 10.1016/J.NEUROIMAGE.2017.04.064 28457975 PMC5518773

[23] Assaf Y , Basser PJ . Composite hindered and restricted model of diffusion (CHARMED) MR imaging of the human brain. Neuroimage. 2005;27(1):48‐58. 10.1016/J.NEUROIMAGE.2005.03.042 15979342

[24] Miller KL , Alfaro‐Almagro F , Bangerter NK , et al. Multimodal population brain imaging in the UK Biobank prospective epidemiological study. Nat Neurosci. 2016;19(11):1523‐1536. 10.1038/nn.4393 27643430 PMC5086094

[25] Smith S , Alfaro Almagro F , Miller K . UK Biobank Brain Imaging Documentation (Version 1.0). 2015 https://biobank.ctsu.ox.ac.uk/crystal/docs/brain_mri.pdf. Accessed January 29, 2016.

[26] Alfaro‐Almagro F , Jenkinson M , Bangerter NK , et al. Image processing and quality control for the first 10,000 brain imaging datasets from UK Biobank. Neuroimage. 2018;166:400‐424. 10.1016/J.NEUROIMAGE.2017.10.034 29079522 PMC5770339

[27] Robertson NL , Moore CM , Ambler G , et al. MAPPED study design: a 6 month randomised controlled study to evaluate the effect of dutasteride on prostate cancer volume using magnetic resonance imaging. Contemp Clin Trials. 2013;34(1):80‐89. 10.1016/j.cct.2012.10.003 23085153

[28] Ahmed HU , Gabe R , Kaplan R , Parker C , Emberton M . Multi‐parametric magnetic resonance imaging in the diagnosis and characterization of prostate cancer prior to first biopsy in men at risk: the PROMIS study. Eur Urol Suppl. 2012;11(1):e824‐e824a. 10.1016/S1569-9056(12)60821-5

[29] Kasivisvanathan V . Magnetic resonance imaging‐targeted biopsy compared to standard trans‐rectal ultrasound guided biopsy for the diagnosis of prostate cancer in men without prior biopsy. WHO—International Clinical Trials Registry Platform. 10.1186/ISRCTN18440098

[30] Bonet‐Carne E , Daducci A , Johnston E , et al. VERDICT prostate parameter estimation with AMICO In: Kaden E, Grussu F, Ning L, Tax C, Veraart J, eds. Computational Diffusion MRI. Mathematics and Visualization. Cham, Switzerland: Springer; 2017:229‐241 10.1007/978-3-319-73839-0_18.

[31] Murday JS , Cotts RM . Self‐diffusion coefficient of liquid lithium. J Chem Phys. 1968;48(11):4938‐4945. 10.1063/1.1668160

[32] Tikhonov AN , Arsenin VY . Solution of ill‐posed problems. Math Comput. 1978;32(144):491 10.2307/2006360.

[33] Johnston E , Pye H , Bonet‐Carne E , et al. INNOVATE: a prospective cohort study combining serum and urinary biomarkers with novel diffusion‐weighted magnetic resonance imaging for the prediction and characterization of prostate cancer. BMC Cancer. 2016;16(1):816 10.1186/s12885-016-2856-2. 27769214 PMC5073433

[34] Panagiotaki E , Ianus A , Johnston E , et al. Optimised VERDICT MRI protocol for prostate cancer characterisation. In: Proceedings of the 23rd Meeting of the International Society for Magnetic Resonance in Medicine 2015 2872, Toronto, Ontario, Canada: ISMRM.

[35] Ourselin S , Roche A , Subsol G , Pennec X , Ayache N . Reconstructing a 3D structure from serial histological sections. Image Vis Comput. 2001;19(1/2):25‐31. 10.1016/S0262-8856(00)00052-4.

[36] Ourselin S , Stefanescu R , Pennec X . Robust registration of multi‐modal images: towards real‐time clinical applications. In: Dohi T, Kikinis R, eds. Medical Image Computing and Computer‐Assisted Intervention—MICCAI 2002: 5th International Conference Tokyo, Japan, September 25‐‐28, 2002 Proceedings, Part II. Berlin, Germany: Springer; 2002:140–147. 10.1007/3-540-45787-9_18.

[37] Dickinson L , Ahmed HU , Allen C , et al. Scoring systems used for the interpretation and reporting of multiparametric MRI for prostate cancer detection, localization, and characterization: could standardization lead to improved utilization of imaging within the diagnostic pathway? J Magn Reson Imaging. 2013;37(1):48‐58. 10.1002/jmri.23689 22566285

[38] Dikaios N , Punwani S , Hamy V , et al. Noise estimation from averaged diffusion weighted images: can unbiased quantitative decay parameters assist cancer evaluation? Magn Reson Med. 2014;71(6):2105‐2117. 10.1002/mrm.24877 23913479 PMC4282362

[39] Gudbjartsson H , Patz S . The Rician distribution of noisy MRI data. Magn Reson Med. 1995;34(6):910‐914. 10.1002/mrm.1910340618 8598820 PMC2254141

[40] Cook PA , Bai Y , Nedjati‐Gilani S , et al. Camino: open‐source diffusion‐MRI reconstruction and processing. Proc Int Soc Magn Reson Med. 2006;14:2759.

[41] Bland JM , Altman DG . Statistical methods for assessing agreement between two methods of clinical measurement. Lancet. 1986;1(8476):307‐310. http://www.ncbi.nlm.nih.gov/pubmed/2868172. Accessed August 2, 2018 2868172

[42] Bonet‐Carne E , Daducci A , Panagiotaki E , et al. Non‐invasive quantification of prostate cancer using AMICO framework for VERDICT MR. In: *Proceedings of the ISMRM 24th Annual Meeting & Exhibition* Singapore:ISMRM; 2016:3465. http://www.camino.org.uk. Accessed August 13, 2018.

[43] Jian B , Vemuri BC . A unified computational framework for deconvolution to reconstruct multiple fibers from diffusion weighted MRI. IEEE Trans Med Imaging. 2007;26(11):1464‐1471. 10.1109/TMI.2007.907552 18041262 PMC2572690

[44] McNeal JE . Normal histology of the prostate. Am J Surg Pathol. 1988;12(8):619‐633. 2456702 10.1097/00000478-198808000-00003

[45] Kaden E , Kelm ND , Carson RP , Does MD , Alexander DC . Multi‐compartment microscopic diffusion imaging. Neuroimage. 2016;139:346‐359. 10.1016/j.neuroimage.2016.06.002 27282476 PMC5517363

[46] Russo G , Mischi M , Scheepens W , De La Rosette JJ , Wijkstra H . Angiogenesis in prostate cancer: onset, progression and imaging. BJUI. 2012;110(11c):E794‐E808. 10.1111/j.1464-410X.2012.11444.x 22958524

[47] Tretiakova M , Antic T , Binder D , et al. Microvessel density is not increased in prostate cancer: digital imaging of routine sections and tissue microarrays. Hum Pathol. 2013 10.1016/j.humpath.2012.06.009 23069258

[48] van Niekerk CG , van der Laak JAWM , Hambrock T , et al. Correlation between dynamic contrast‐enhanced MRI and quantitative histopathologic microvascular parameters in organ‐confined prostate cancer. Eur Radiol. 2014 10.1007/s00330-014-3301-z 25033819

[49] Lissbrant IF , Stattin P , Damber JE , Bergh A . Vascular density is a predictor of cancer‐specific survival in prostatic carcinoma. Prostate. 1997 10.1002/(SICI)1097-0045(19970915)33:1<38::AID-PROS7>3.0.CO;2-5 9294625

[50] Rubin MA , Buyyounouski M , Bagiella E , et al. Microvessel density in prostate cancer: lack of correlation with tumor grade, pathologic stage, and clinical outcome. Urology. 1999 10.1016/S0090-4295(98)00561-5 10096381

[51] Miyata Y , Sakai H . Reconsideration of the clinical and histopathological significance of angiogenesis in prostate cancer: usefulness and limitations of microvessel density measurement. Int J Urol. 2015 10.1111/iju.12840 26153072

[52] Bourne RM , Bailey C , Johnston EW , et al. Apparatus for histological validation of in vivo and ex vivo magnetic resonance imaging of the human prostate. Front Oncol. 2017;7:47 10.3389/fonc.2017.00047 28393049 PMC5364138

[53] Sabouri S , Chang SD , Savdie R , et al. Luminal water imaging: a new MR imaging T2 mapping technique for prostate cancer diagnosis. Radiology. 2017;284(2):451‐459. 10.1148/radiol.2017161687 28394754 PMC5522021

